# QCM Biosensor Based on Polydopamine Surface for Real-Time Analysis of the Binding Kinetics of Protein-Protein Interactions

**DOI:** 10.3390/polym9100482

**Published:** 2017-10-02

**Authors:** Chunli Wu, Xueming Li, Siyu Song, Yuxin Pei, Lili Guo, Zhichao Pei

**Affiliations:** 1 School of Pharmaceutical Sciences, Zhengzhou University, 100 Kexue Avenue, Zhengzhou 450001, China; kedi2009@126.com; 2 Shaanxi Key Laboratory of Natural Products & Chemical Biology, College of Chemistry & Pharmacy, Northwest A & F University, Yangling 712100, China; lixueming1988@163.com (X.L.); ssyu137@163.com (S.S.); peiyx@nwafu.edu.cn (Y.P.); 18337149831@163.com (L.G.)

**Keywords:** polydopamine, quartz crystal microbalance, biosensor, protein-protein interactions, binding kinetics

## Abstract

A quartz crystal microbalance (QCM) biosensor based on polydopamine (PDA) surface was developed for real-time analysis of the binding kinetics of protein-protein interactions. The biosensor was fabricated by simply immersing the gold sensor chip into an aqueous dopamine solution at pH 8.5 leading to a spontaneous deposition of PDA film onto the sensor chip surface, which was followed by incubation with the protein to immobilize it onto the PDA-coated sensor chip surface via Michael addition and/or Schiff base reactions. In this paper, the interaction between monoclonal anti-myoglobin 7005 antibody (IgG1) and its antigen human cardiac myoglobin was used as a model system for real-time analysis of biomolecule interactions on the biosensor surface. The kinetic parameters of the interaction between anti-myoglobin 7005 and myoglobin were studied on the biosensor surface, which were consistent with the results obtained via amine coupling. The biosensor based on PDA surface has excellent regenerability, reproducibility, and specificity. Compared with the most frequently/typically used amine coupling method for immobilization of proteins on carboxylated substrates, the modification methodology presented in this paper is simple, mild and is not subjected to the limitations of the isoelectric point (pI) of the protein. In addition, the PDA biosensor chip can be easily reused, which makes QCM biosensor analysis more efficient and cost effective.

## 1. Introduction

In recent years, the quartz crystal microbalance (QCM) based upon the piezoelectric effect has proved to be a powerful and efficient tool for real-time and label-free evaluation of biomolecular interactions [1,2,3,4]. Biosensors based on QCM technology have not only been used alone but have also been widely used in combination with other techniques [5] such as surface plasmon resonance (SPR) [6,7,8,9,10], localized surface plasmon resonance (LSPR) [11,12,13,14], ellipsometry [15,16], reflectometry [17,18,19], for clinical diagnosis, drug discovery, food safety, environmental monitoring, and academic research [20,21,22,23,24,25].

**Figure 1:**
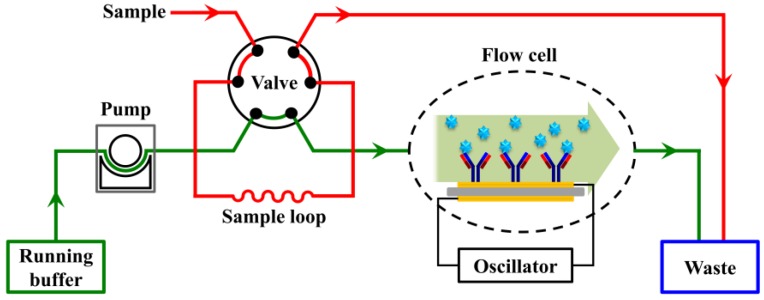
Schematic of the QCM (quartz crystal microbalance) biosensor flow-through system.

The typical QCM instrument consists of a flow-through system where the buffer continuously flows over the sensor surface in the flow cell. The analyte is diluted in the buffer and then introduced onto the sensor surface, where the ligand has been immobilized, via a six-way valve. The frequency shift resulting from the interaction between the analyte and the ligand is monitored in real-time (Figure 1) [26,27,28]. To study the interaction between a pair of biomolecules, one of the pair (the ligand) needs to be first immobilized onto the sensor surface, which is generally done by exploiting either noncovalent or covalent reactions. Noncovalent methods normally rely on physical adsorption and affinity capture, such as adsorption on hydrophobic surface [29,30] and capture via antibody-antigen [31], streptavidin-biotin [32], and nitriloacetic acid (NTA)-histidine [28] or DNA-DNA [33] interactions. In contrast, covalent immobilization typically relies on the conjugation reactions of active functional groups, such as amine coupling [34], aldehyde coupling [30], and thiol coupling [35]. These strategies usually require complicated processes. For example, in the commonly used surface modification processes, the sensor surface is first functionalized with alkanethiol agents with carboxyl groups by forming self-assembled monolayer (SAM). Then, the carboxylated sensor surfaces is activated using 1-ethyl-3-(3-(dimethylamino)propyl)carbodiimide (EDC) and *N*-hydroxysuccinimide (NHS). Proteins such as antibodies are covalently attached to the active surface via amine coupling. Remaining active groups on the surface are then deactivated using ethanolamine [36]. The hydrolysis of the active NHS-esters during reaction and storage lead to low efficiency of the immobilization [37], and the chemicals used for said surface modifications are generally expensive or require many steps to synthesize. In addition, the successful immobilization via amine coupling is highly dependent on the surface p*K*a, antibody pI, and pH of the immobilization buffer [38]. Acidic proteins with pI lower than 3.5 generally cannot be attached using the amine coupling method [39]. Recently, Velotta and coworkers demonstrated the effectiveness of ultrashort UV laser pulse in breaking the disulfide bridge in antibody to produce highly reactive thiol groups, which can efficiently link the gold surfaces of a QCM [40,41].

Polymers are also used for biomolecule immobilizations in noncovalent or covalent strategies, such as physical adsorption on a polystyrene coated surface [29,30], covalent conjugation on a flexible hydrogel matrix composed of carboxymethylated dextran [42] or dynamic polymers based on biotinylated poly(acrylic acid) brushes [43]. Although very easy to realize, the physical adsorption on hydrophobic surfaces is not robust, as it is affected by many physical and chemical interactions between the protein, surface and solvent [44,45]. On the other hand, covalent conjugation on polymers enables the stable immobilization of biomolecules, but normally requires multiple steps of modification and strictly controlled reaction conditions, which makes the immobilization process time-consuming and inefficient [42,43,46].

Inspired by the composition of adhesive proteins in mussels, polydopamine (PDA) coatings can be formed via dopamine self-polymerization in aqueous solution, which then can be deposited onto virtually any type of inorganic and organic materials through simply dip-coating objects in an aqueous solution of dopamine [47,48,49,50]. The catechol/quinone groups present in PDA coating can react with nucleophilic amine or thiol groups via Michael addition and/or Schiff base reactions to conjugate the amine and/or thiol-containing molecules onto the PDA surface for secondary modifications [37,47,51].

In this study, a QCM biosensor based on polydopamine (PDA) surface was developed for real-time analysis of the binding kinetics of protein-protein interactions. The sensor surface was fabricated through a simple process containing two steps of incubation treatments under mild conditions. The regenerability, reproducibility, and specificity of the sensor were also evaluated. The interaction between anti-myoglobin 7005 antibody and myoglobin antigen was used as a typical example for real-time analysis of the binding kinetics of protein-protein interactions on this biosensor surface. A comparison of the kinetic parameters obtained on a carboxyl sensor surface, where anti-myoglobin 7005 antibody was immobilized via amine coupling, was also performed. To the best of our knowledge, this is the first time that the PDA platform and QCM biosensor were combined to analyse the binding kinetics of protein-protein interactions in real time.

## 2. Materials and Methods

### 2.1. Materials

Dopamine hydrochloride, tris(hydroxymethyl)aminomethane (Tris), BSA and fluorescein isothiocyanate-conjugated avidin (avidin-FITC) were purchased from Sigma (Shanghai, China). 1-Ethyl-3-(3-dimethyl-aminopropyl)carbodiimide hydrochloride (EDC), *N*-hydroxysulfosuccinimide (sulfo-NHS) and ethanolamine (1 M, pH 8.5) were obtained from Attana (Stockholm, Sweden). Monoclonal anti-myoglobin 7005 antibody (IgG1) was purchased from Medix Biochemica (Kauniainen, Finland). Human cardiac myoglobin was purchased from BiosPacific (Emeryville, CA, USA). Other reagents were of analytical-reagent grade purchased from local suppliers, and used without further purification. Attana gold and LNB-carboxyl sensor chips (Attana, Stockholm, Sweden) with 10 MHz AT-cut quartz crystals were used in this study. The quartz disc is round shape with a diameter of 8 mm, on each side of which gold electrodes are plated with a diameter of 4.5 mm. The sensor chip is mounted in the flow cell (1.4 μL, with a chamber height of 50 μm and a diameter of 6 mm) by an O-ring so that only one side of the chip surface is exposed to the continuous flow of solutions.

### 2.2. PDA Coating

The PDA sensor chip was fabricated on an Attana gold sensor chip. The gold chip was cleaned using piranha solution (conc. H_2_SO_4_:30% H_2_O_2_ = 3:1 *v*/*v*) for 30 min, followed by thorough rinsing with water and drying under a stream of nitrogen. (Caution: Piranha solution reacts violently with organic solvents. Use extreme precautions when handling this solution.) Then, a drop of 50 μL dopamine solution (2 mg/mL) in Tris buffer (10 mM, pH 8.5) was applied to the cleaned gold chip surface and incubated in humid environment overnight. After incubation, the sensor chip was rinsed with water and dried under a stream of nitrogen.

### 2.3. Contact Angle Measurement

The wettability of the sensor surface before and after PDA coating was characterized on the basis of static contact angle measurement using a water contact angle meter (SL100, Solon Information Technology Co., Ltd., Shanghai, China). At least three measurements on three different places were taken and averaged to get a reliable value for each sensor chip.

### 2.4. X-ray Photoelectron Spectroscopy (XPS) Measurement

XPS spectra were obtained using a Thermo Scientific K-Alpha instrument (Thermo Fisher Scientific Inc., Waltham, MA, USA) to determine the chemical composition of the sensor surface.

### 2.5. Protein Immobilization

A drop of 50 μL protein solution at 100 μg/mL diluted in PBS buffer (pH 7.4) was added onto the PDA coated sensor chip surface and incubated for 4 h at 4 °C in humid environment. Following incubation, the sensor surface was washed with PBS buffer to remove un-immobilized proteins. Thereafter, the sensor chip was mounted in a chip holder and docked into the Attana Cell A200 QCM instrument (Attana, Stockholm, Sweden) for real-time measuring of the interactions between the immobilized protein and its interacting protein. The short range term (1600 s) the stability of the quartz oscillator was described in Figure S1.

### 2.6. QCM Analysis of Protein-Protein Interactions

All interaction measurements were performed using an Attana Cell A200 QCM biosensor instrument. The sensor chip was docked into the instrument and stabilized under a continuous flow (25 μL/min) of PBS running buffer (10 mM, pH 7.4). When the baseline is stable (the frequency drift being less than 0.2 Hz/min), the interaction measurements can be started. A 50 μL sample loop was used, and only 35 μL was injected to diminish dispersion. The analyte diluted in running buffer was injected over the sensor chip surface, allowing 84 s for association and 300 s for dissociation. The frequency shift (Δ*f*) resulting from the association and dissociation of the analyte on the sensor chip surface was recorded using the Attester software (Attana, Stockholm, Sweden) in real time. Following each association and dissociation cycle, the sensor chip surface was regenerated by an injection of 10 mM glycine (pH 1.5) to desorb the bound analyte. The data was analyzed using the evaluation software provided with the Attana Cell A200 instrument, where the kinetic analysis was performed by global fitting the sensorgram curves using a theoretical 1:1 interaction model with mass transport limitation. The dissociation constant was calculated using the equation *K*_D_ = *k*_diss_/*k*_ass_.

### 2.7. Reuse of the Sensor Chip

After the QCM measurement, the PDA-coating on the sensor chip was degraded according to the literature [52] by immersing the sensor chip in 1 g/L sodium hypochlorite (NaClO) solution for 5 min followed by extensive rinsing with water. Thereafter, the chip could be reused for further PDA-coating as described in Section 2.2.

## 3. Results and Discussion

**Figure 2:**
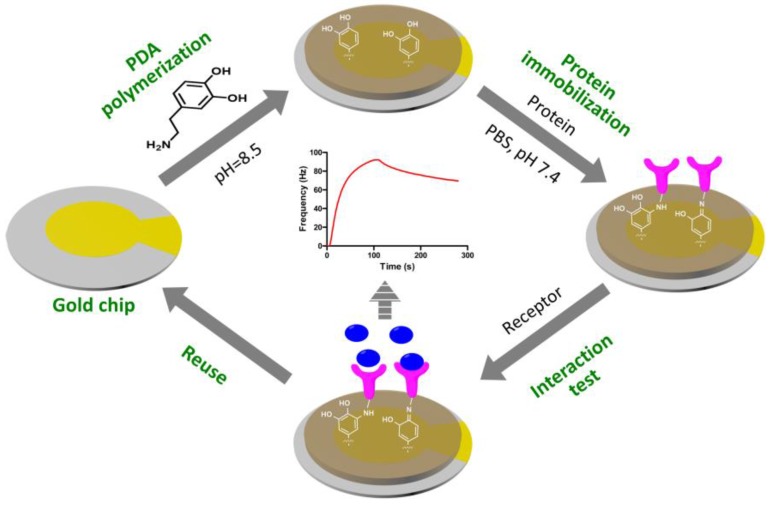
Schematic illustration of the QCM biosensor based on polydopamine surface.

The QCM biosensor based on PDA surfaces was fabricated as shown in Figure 2. Firstly, the cleaned sensor chip surface was coated with PDA film via the self-polymerization of dopamine in alkaline solution. Then, the protein was immobilized onto the PDA-coated sensor surface via Michael addition and/or Schiff base reactions. The interaction between the immobilized protein and its target was monitored using QCM biosensor instruments in real-time. The sensor chip fabricated via this simple procedure could be easily reused, which would make the QCM biosensor analysis more efficient and cost effective.

### 3.1. PDA Coating

**Figure 3:**
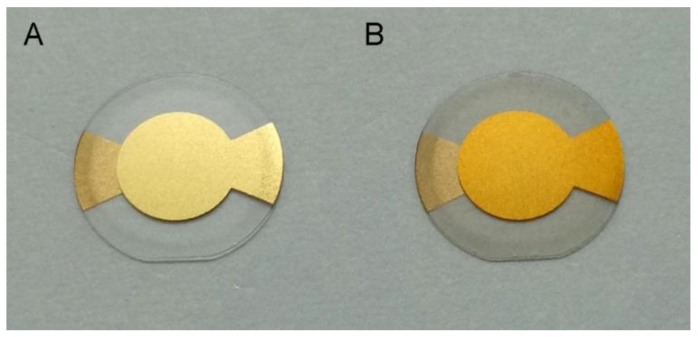
The QCM gold sensor chip before (**A**) and after (**B**) PDA (polydopamine) coating.

**Figure 4:**
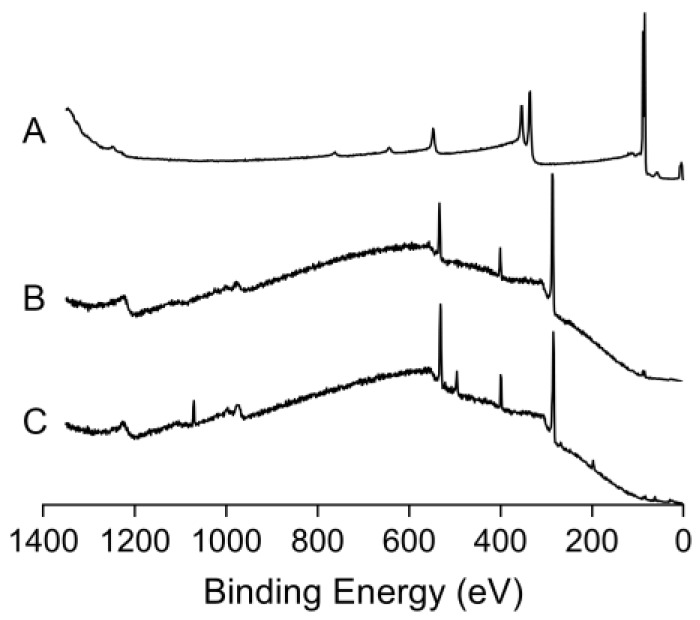
XPS (X-ray photoelectron spectroscopy) spectra of unmodified gold sensor chip (**A**), PDA-coated gold sensor chip (**B**) and anti-myoglobin 7005 antibody immobilized PDA coated gold sensor chip (**C**).

The PDA-coated sensor chip surface was fabricated by simply incubating the cleaned gold sensor chip with a drop of the alkaline aqueous solution of 2 mg/mL dopamine, where the PDA deposits onto the sensor chip surface through self-polymerization of dopamine. After the incubation, the color of the gold sensor chip turned from yellow to brown (Figure 3). The water contact angle of the sensor chip surface changed from 67° to 48°, which is in accordance with a previous report [47]. The measured frequency shift due to PDA coating was determined to be 1841 ± 44 Hz (mean ± S.D., *n* = 5), resulting in a corresponding film mass of 1.29 ± 0.03 μg according to the Sauerbrey equation (Equation (1)) [29,53]. X-ray photoelectron spectroscopic (XPS) analysis of the gold sensor chip before and after PDA-coating (Figure 4A,B) shows the suppression of photoelectron peaks of Au (84 eV for Au4f7, 88 eV for Au4f5, 336 eV for Au4d5, 354 eV for Au4d3 and 547 eV for Au4p3), along with the emergence of carbon peak (C1s, 285 eV), oxygen (O1s, 531 eV) and nitrogen peak (N1s, 400 eV). The nitrogen-to-carbon ratio (N/C) of the PDA-coated gold sensor chip surface (N/C = 0.116) is similar to the theoretical value for dopamine (N/C = 0.125) and the reported value for PDA coating [54]. These results indicate the successful coating of PDA onto the gold sensor chip surface.

(1) $$\Delta f=-\frac{{2f}_{0}^{2}}{A\sqrt{\rho_{q}\mu_{q}}}\Delta m$$

*f*_0_—Resonant frequency (*f*_0_ = 10.0 MHz), ∆*f*—Frequency change (Hz), ∆*m*—Mass change (g), A—Piezoelectrically active crystal area (Area between electrodes, 0.159 cm^2^), *ρ*_q_—Density of quartz (*ρ*_q_ = 2.648 g/cm^3^), *μ*_q_—Shear modulus of quartz for AT-cut crystal (*μ*_q_ = 2.947 × 10^11^ g·cm^−1^·s^−2^).

### 3.2. Protein Immobilization on the PDA-Coated Sensor Chip Surface

**Figure 5:**
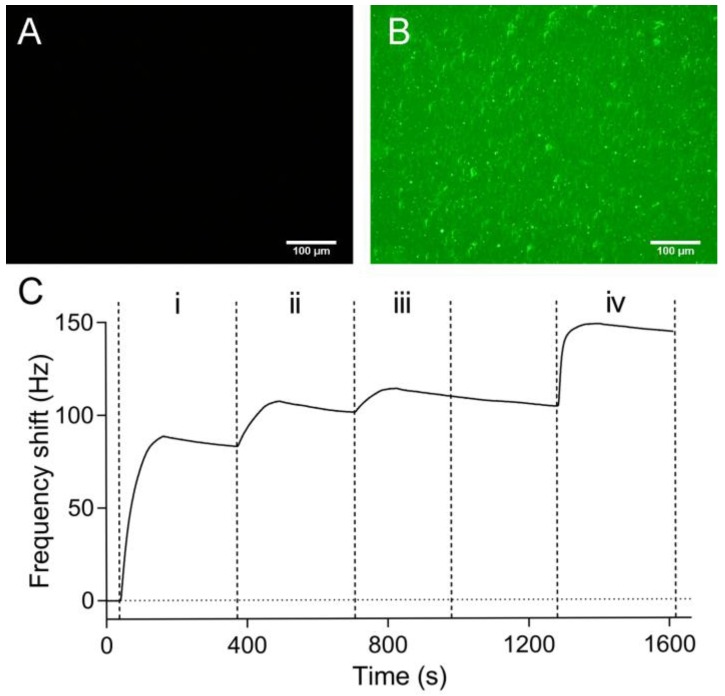
Fluorescent images of the PDA-coated sensor chip before (**A**) and after (**B**) the incubation with avidin-FITC (fluorescein isothiocyanate) (**C**) The subsequent biotinylated Con A capturing and mannan test on the avidin-FITC surface were performed by three injections of 100 μg/mL biotinylated Con A (i–iii) and an injection of 100 μg/mL mannan (iv).

The many functional groups present in the PDA coating are able to react with a wide range of molecules [37,55,56]. Among them, the most widely investigated reactions are the covalent coupling reaction of the catechol/quinone groups in PDA with the amine and/or thiol containing molecules via Michael addition and/or Schiff base reactions [37,48]. In this study, proteins were directly immobilized onto the PDA-coated QCM sensor chip surface by simply immersing the chip in a protein solution at pH 7.4 for several hours. As shown in Figure 5A,B, after the immobilization of avidin-FITC onto the PDA-coated sensor chip surface, green fluorescence could be observed. Then the chip was docked into the Attana Cell A200 QCM biosensor system and equilibrated with PBS running buffer. When the base line was stable (the drift being less than 0.2 Hz/min), biotinylated Con A was subsequently captured on to the sensor surface through three injections of 100 μg/mL biotinylated Con A, resulting in the frequency shift of 104 Hz. The decay may has been caused by the impurity in biotinylated Con A, which can produce non-specific binding. It will become stable with longer time when the loose non-specific binding was removed with continuous flow buffer solution (Figure S2). Mannan was tested on the surface by an injection of 100 μg/mL mannan giving a response of 44 Hz (Figure 5C). These results confirmed the successful immobilization of the avidin-FITC onto the PDA-coated sensor chip surface.

In immunosensor sciences, the most frequently used amine coupling methods for immobilization of antibodies onto the sensor surface normally require complex steps and strictly controlled reaction conditions [38,39]. In addition, the immobilization efficiency is highly dependent on the surface p*K*a, antibody pI, and pH of immobilization buffer, owing to the electrostatic attraction effect between the surface and the antibody [38]. Acidic proteins with pI lower than 3.5 generally cannot be attached using amine coupling method [39]. For the monoclonal anti-myoglobin 7005 antibody, which has a pI of 5.9–6.4, the immobilization is effective only when the pH of immobilization buffer is below 5.0. Above pH 5.0, the immobilization is highly sensitive to small increases in pH, which can cause dramatic decreases in immobilization efficiency [38]. In this study, the immobilization of anti-myoglobin 7005 antibody onto PDA coating surface was easily achieved under mild conditions (in PBS, pH 7.4) for real-time evaluation of the binding kinetics of antibody-antigen interactions using QCM technology. From the analysis of XPS, it was found that the immobilization of anti-myoglobin 7005 antibody on the PDA coated sensor chip surface altered obviously the surface chemical composition as measured by increases in the peaks corresponding to nitrogen and oxygen and decreases in the carbon peak (Figure 4C). The N/C ratio for the PDA-coated sensor surface changed from 0.116 to 0.169 after the immobilization of anti-myoglobin 7005 antibody. The measured frequency shift due to anti-myoglobin 7005 antibody immobilization was determined to be 455 ± 45 Hz (mean ± S.D., *n* = 3), resulting in a corresponding mass of 0.32 ± 0.03 μg according to the Sauerbrey equation [29,53]. Compared with the most frequently used amine coupling method for immobilization of proteins on carboxylated substrates, the modification methodology presented in this paper is simple, mild and is not limited by the pI of the protein.

### 3.3. QCM Measurements of Protein-Protein Interaction

**Figure 6:**
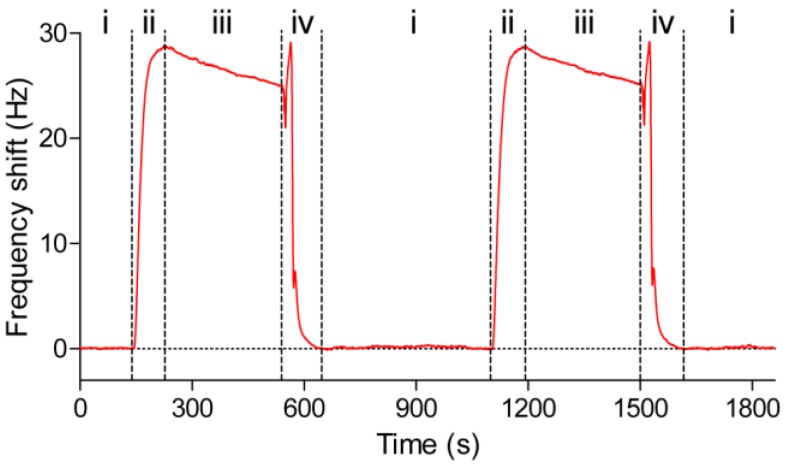
QCM analysis of the binding of myoglobin with anti-myoglobin 7005 antibody immobilized on the PDA-coated sensor surface. Frequency shift was recorded during the association (84 s) and dissociation (300 s) phases of the interaction between myoglobin and anti-myoglobin 7005, as well as the subsequent desorption of the remaining bound myoglobin by an injection of the 10 mM glycine pH 1.5. (i) Base line; (ii) Association; (iii) Dissociation; (iv) Regeneration.

**Figure 7:**
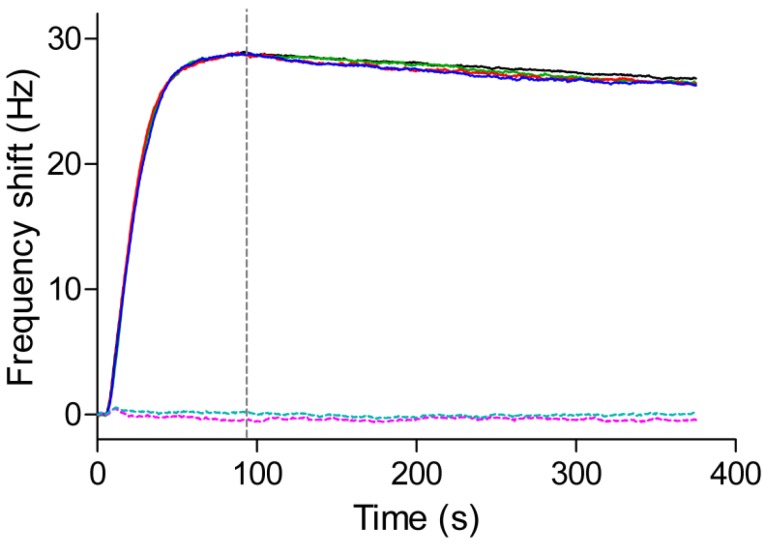
Reproducibility and specificity of the sensor surface. Solid lines: four cycles (black, red, green and blue curves) of the interaction between myoglobin (4 μg/mL) and anti-myoglobin 7005 antibody; Dotted lines: two cycles (teal and magenta curves) of the interaction between BSA (4 μg/mL) and anti-myoglobin 7005 antibody.

The QCM sensor chip, consisting of anti-myoglobin 7005 antibodies immobilized on a PDA-coated senor chip surface, was docked into an Attana Cell A200 QCM biosensor and equilibrated under a continuous flow (25 μL/min) of PBS running buffer. When the resonant frequency was stable (baseline drift less than 0.2 Hz/min), an injection consisting of 4 μg/mL myoglobin antigen was performed to evaluate the interactions between the immobilized anti-myoglobin 7005 antibody and the myoglobin antigen. Following each injection and measurement, a 30 s pulse of 10 mM glycine pH 1.5 was introduced to the sensor surface to desorb the remaining myoglobin. The interaction and regeneration processes were monitored in real time using the Attester software. Figure 6 shows a typical sensorgram in which two injections of myoglobin at 4 μg/mL were performed successively with regeneration steps after each measurement. When the myoglobin was introduced onto the sensor surface, the signal increased as a result of the binding between the myoglobin and the anti-myoglobin 7005 antibody. After 84 s, the pure buffer without the myoglobin was allowed to flow over the sensor surface, the dissociation between the myoglobin and anti-myoglobin 7005 antibody led to a decrease in the signal. After an injection of the regeneration solution consisting of 10 mM glycine at pH 1.5, the frequency restored to the base line, which indicated that all of the remaining bound myoglobin had been desorbed from the sensor surface. The sensor surface was then ready for the next cycle of interaction measurement. Figure 7 shows the superimposed binding curves of four cycles of the antigen-antibody interaction with the regeneration step in between. The high degree of overlap between the curves further indicates a high degree of reproducibility of the sensor surface. The null-control protein BSA yielded little response, and have no effect on the interaction between myoglobin and anti-myoglobin 7005 antibody (Figure 7 and Figure S3), confirming the specificity of the binding event on the sensor chip surface.

### 3.4. Kinetic and Affinity Studies

**Figure 8:**
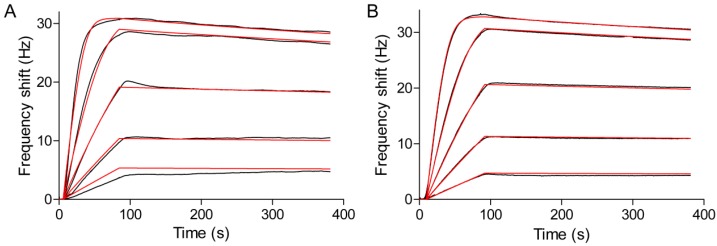
Kinetic evaluation of the interactions between myoglobin and anti-myoglobin 7005 antibody. Myoglobin at 0.25, 0.5, 1, 2 and 4 μg/mL (14.7, 29.4, 58.8, 118 and 235 nM) was injected over the sensor surface and the responses were recorded (black lines). Theoretical 1:1 fit using the Evaluation software (Attana) was overlaid (red lines). (**A**) Anti-myoglobin 7005 antibody was immobilized on the PDA-coated sensor surface; (**B**) Anti-myoglobin 7005 was immobilized on the carboxyl sensor surface via amine coupling.

The real-time in situ measurement of the binding kinetics of the interaction between myoglobin and the immobilized anti-myoglobin 7005 antibody was performed on the PDA-coated sensor surface using the Attana Cell A200 QCM biosensor. Before every injection of the myoglobin, the running buffer was injected over the surface as a reference. A serial dilution concentration series of myoglobin diluted in running buffer ranging from 0.25 to 4 μg/mL were injected over the surface respectively with regeneration steps after each measurement. The binding was monitored in real time and the binding responses are shown in Figure 8A. The global fit using the theoretical 1:1 interaction model with mass transport limitation was performed using the Evaluation software provided with the Attana Cell A200 instrument. The kinetic rate constants of the interaction between anti-myoglobin 7005 antibody and myoglobin, such as the association rate constant (*k*_ass_ = 6.10 × 10^5^ M^−1^·s^−1^) and dissociation rate constant (*k*_diss_ = 3.39 × 10^−4^ s^−1^), were obtained. From these values, the dissociation constant (*K*_D_ = 0.556 nM) was calculated using the ratio of the binding rate constants (*K*_D_ = *k*_diss_/*k*_ass_). To compare the results, kinetic evaluation of the interaction between anti-myoglobin 7005 antibody and myoglobin on a carboxyl sensor surface, where anti-myoglobin 7005 antibody was immobilized via amine coupling, was performed (Figure 8B). The obtained kinetic and affinity results (*k*_ass_ = 5.27 × 10^5^ M^−1^·s^−1^, *k*_diss_ = 2.83 × 10^−4^ s^−1^ and *K*_D_ = 0.573 nM) strongly correlated with the results obtained on the PDA-coated sensor surface, verifying the performance of the QCM biosensor based on PDA surface.

### 3.5. Reuse of the Sensor Chip for Knietic and Affinity Studies

**Figure 9:**
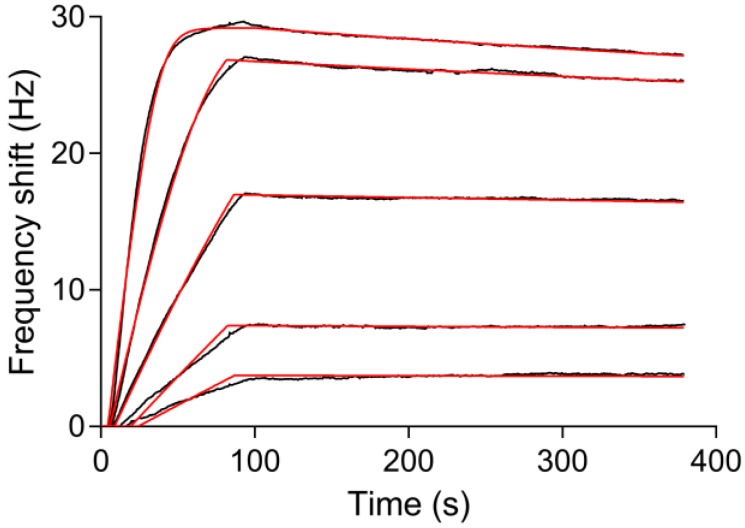
Reuse of the sensor chip for kinetic evaluation of the interactions between myoglobin and anti-myoglobin 7005 antibody. Myoglobin at 0.25, 0.5, 1, 2 and 4 μg/mL (14.7, 29.4, 58.8, 118 and 235 nM) was injected over the sensor surface and the responses were recorded (black lines). Theoretical 1:1 fit using the Evaluation software (Attana) was overlaid (red lines).

In immunosensor sciences, the sensor chip is the main and most costly consumable for biomolecule interaction assays. Cyclic use of a single sensor chip in several determinations of various analytical samples would make biosensor assays more cost effective. A wide range of strategies have been devised in order to reuse sensor chip platforms. Normally, the sensor surface is regenerated by breaking the biomolecular interactions between antigen and antibody using high salt, acidic or alkaline solutions [57,58,59]. Through these methods, the sensor chip can only be reused for a limited number of times due to the inactivation of the immobilized biomolecules. It is also feasible to completely regenerate the gold surface [60], but this requires complicated re-fabrication of the functionalized sensor surface, leading to low efficiency of the biosensor assays. There are also some capture strategies, such as His-tag capture [28], that enable the reversible immobilization of biomolecules. However, they normally need complex procedures and special conditions. The PDA coated sensor chip fabricated in this study can be easily reused by simply immersing the sensor chip into a NaClO solution for several minutes to degrade the PDA-coating, which restores the initial gold sensor chip surface [52]. Then, the sensor chip can be reused for the next cycle of PDA-coating, protein immobilization and interaction measurements. Figure 9 shows the reuse of the sensor chip for kinetic evaluation of the interactions between myoglobin and anti-myoglobin 7005 antibody. The obtained association rate constant (*k*_ass_ = 6.79 × 10^5^ M^−1^·s^−1^), dissociation rate constant (*k*_diss_ = 2.94 × 10^−4^ s^−1^) and dissociation constant (*K*_D_ = 0.434 nM) are similar to the results in Section 3.4. The simple procedure for fabrication and reuse of the sensor chip presented in this study would make QCM biosensor analyses more efficient and cost effective.

## 4. Conclusions

In this study, a QCM biosensor based on PDA surface was developed for real-time analysis of the binding kinetics of protein-protein interactions. The PDA-coated sensor surface was prepared through an extremely simple incubation process where a drop of alkaline dopamine solution was applied onto the gold sensor chip surface, with the PDA depositing onto the chip surface through self-polymerization of dopamine. Proteins were directly attached to the PDA-coated sensor surface via Michael addition and/or Schiff base reactions without the use of any coupling agent or pre-treatment. The fabricated sensor chip based on PDA surface exhibited excellent regenerability, reproducibility and specificity, which are important features for reliable real-time evaluation of the binding kinetics of protein-protein interactions. The kinetic results obtained using this method strongly correlated with the results obtained via amine coupling, verifying the successful combination of PDA platform and QCM biosensor for binding kinetic studies. Compared with the typically used amine coupling methods for immobilization of proteins on carboxylated substrates, the modification methodology presented in this paper is simple, mild and is not limited by the pI of the protein. In addition, the simple procedures for the fabrication as well as reuse of the sensor chip will make QCM biosensor analyses more efficient and cost effective.

## Supplementary Materials

The following are available online at www.mdpi.com/2073-4360/9/10/482/s1. Figure S1: The short range term (1600 s) the stability of the quartz oscillator, Figure S2: The sensorgram of two injections of 100 μg/mL biotinylated Con A on the avidin-FITC surface, Figure S3: The interactions between myoglobin (4 μg/mL) and anti-myoglobin 7005 antibody without (red line) or in the presence of (black line) BSA (4 μg/mL).
