# Supplementary material for: QCM Biosensor Based on Polydopamine Surface for Real-Time Analysis of the Binding Kinetics of Protein-Protein Interactions
Source: Polymers (Basel). 2017 Oct 2;9(10):482. doi: 10.3390/polym9100482 (PMC6418727; doi:10.3390/polym9100482)
Supplement: Supplementary file 1 [file polymers-09-00482-s001.pdf]

Supplementary Materials: QCM biosensor based on polydopamine surface for real-time analysis of the binding kinetics of protein-protein interactions

Chunli Wu, Xueming Li, Siyu Song, Yuxin Pei, Lili Guo and Zhichao Pei

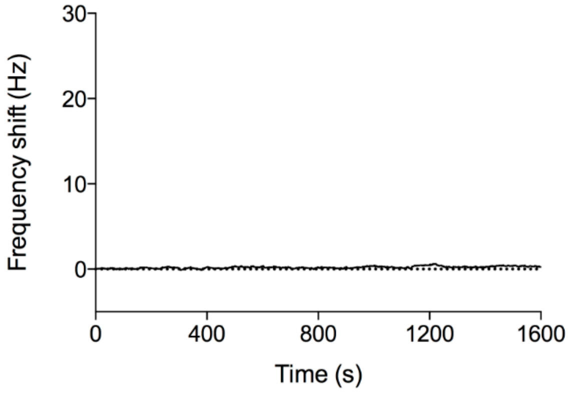

Figure S1. The short range term (1600 s) the stability of the quartz oscillator.

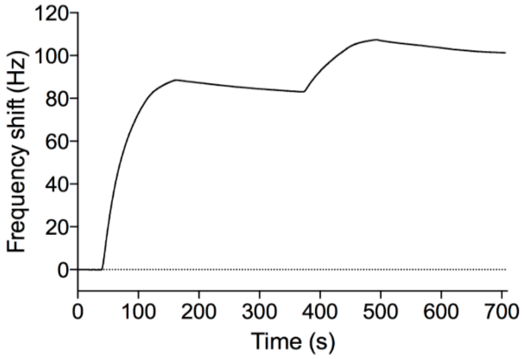

Figure S2. The sensorgram of two injections of 100 μg/mL biotinylated Con A on the avidin-FITC surface.

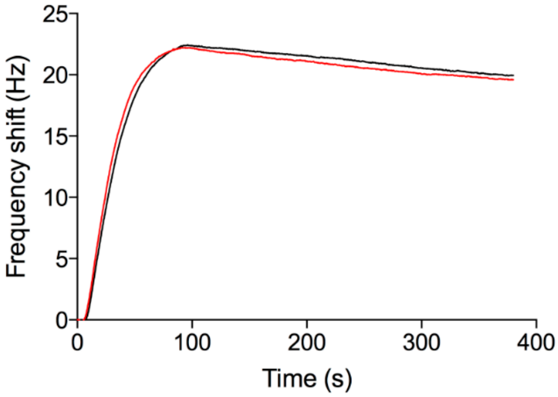

Figure S3. The interactions between myoglobin (4 μg/mL) and anti-myoglobin 7005 antibody without (red line) or in the presence of (black line) BSA (4 μg/mL).
